# Nonalcoholic fatty liver disease remission in men through regular exercise

**DOI:** 10.3164/jcbn.17-115

**Published:** 2018-03-17

**Authors:** Takafumi Osaka, Yoshitaka Hashimoto, Masahide Hamaguchi, Takao Kojima, Akihiro Obora, Michiaki Fukui

**Affiliations:** 1 Department of Endocrinology and Metabolism, Kyoto Prefectural University of Medicine, Graduate School of Medical Science, 465 Kajii-cho, Kawaramachi-Hirokoji, Kamigyo-ku, Kyoto 602-8566, Japan; 2 Department of Diabetology, Kameoka Municipal Hospital, 1-1 Shinoda Shino-cho, Kameoka, Kyoto 621-8585, Japan; 3 Department of Gastroenterology, Murakami Memorial Hospital, Asahi University, 3-2-3 Hashimoto-cho, Gifu 500-8523, Japan

**Keywords:** NAFLD, fatty liver, exercise, remission

## Abstract

Recent cross-sectional and randomized controlled studies of small sample sizes revealed that regular exercise is effective for improving nonalcoholic fatty liver disease. However, there has been no large-scale longitudinal study addressing the effect of regular exercise on remission of nonalcoholic fatty liver disease. Thus, we investigated the impact of exercise on the natural history of nonalcoholic fatty liver disease. We analyzed 1,010 (860 men and 150 women) Japanese participants who received health checkups repeatedly over 10 years by a historical cohort study and were diagnosed with nonalcoholic fatty liver disease at baseline. Regular exercise was defined as participating in any kind of sports at least once a week. Nonalcoholic fatty liver disease was diagnosed by ultrasonographic images. During 10 years of follow-up, remission of nonalcoholic fatty liver disease was observed in 46.0% (396/860) of men and 48.7% (73/150) of women. In men, the adjusted hazard ratio of regular exercise for remission of nonalcoholic fatty liver disease was 1.46 (95% confidence interval 1.10–1.95, *p* = 0.010). However, this was not significant in women. Exercise at least once a week is implicated in the remission of nonalcoholic fatty liver disease in men.

## Introduction

Nonalcoholic fatty liver disease (NAFLD) is not only a major cause of liver-related morbidity and mortality^(1,2)^ but also a risk factor for type 2 diabetes,^(3,4)^ chronic kidney disease^(5)^ and cardiovascular disease.^(6–8)^ In Japan, its prevalence has increased to 20–30% along with the increased consumption of a high-fat diet and the expansion of a sedentary lifestyle.^(9–11)^

Because of the importance of NAFLD, several different strategies have been designed for NAFLD treatment, including pharmacological agents and surgical interventions, but definitive therapy has not been established.^(12)^ It is well known that body weight reduction leads to remission or improvement of NAFLD.^(10,13)^ In addition, there has been increasing interest in lifestyle therapies for NAFLD.^(14)^ Several cross-sectional studies revealed that regular exercise is associated with a lower NAFLD prevalence.^(15–19)^ However, the impact of regular exercise on the natural history of NAFLD remains to be elucidated, because there is no large longitudinal study addressing the effect size of regular exercise on remission of NAFLD. Therefore, in this study we investigated a longitudinal analysis of the effect of regular exercise on the natural history of NAFLD in a large-scale Japanese population using standardized ultrasonographic diagnosis for fatty liver as well as standardized questionnaires for lifestyle.^(20)^

## Materials and Methods

### Study population

We designed a historical cohort study to investigate the impact of regular exercise on the natural history of NAFLD in participants who underwent a medical health checkup program at Murakami Memorial Hospital, Gifu, Japan. This longitudinal analysis was termed NAGALA (NAfld in Gifu Area, Longitudinal Analysis). The purpose of the medical health checkup program and the detailed characteristics of participants were described previously.^(10)^ In the current study, we included the participants who received the health checkups repeatedly over a decade and who had a fatty liver disease at baseline examination. Exclusion criteria were alcohol intake of more than 20 g/day, known liver disease, or current use of any medication.^(10,21,22)^ Participants with known liver disease included those who were positive for hepatitis B antigen or hepatitis C antibody or those with a history of viral, genetic, autoimmune, or drug-induced liver disease.^(23)^ The ethics committee of Murakami Memorial Hospital approved the study, and informed consent was obtained from all participants.

### Data collection

The following tests were performed for the collection of data: urinalysis, blood cell counts, blood chemistry and abdominal ultrasonography. The medical history and lifestyle factors of all participants were surveyed by a standardized self-administered questionnaire. We undertook blood and urine examinations using MODULAR ANALYTICS (Hitachi High-Technologies Corp. Ltd., Tokyo, Japan). Body mass index (BMI) was calculated as body weight in kilograms divided by the participant’s height in meters squared.

### Standardized questionnaire for lifestyle factors

A standardized questionnaire was administered to all participants by the same trained team of interviewers. Alcohol consumption was evaluated by asking the participants for the amount and type of alcoholic beverages consumed per week during the past month, then estimating the mean ethanol intake per week and the total amount of alcohol consumed per week, in grams.^(24)^ The validity of alcohol consumption data was confirmed previously.^(10)^ Smoking status was categorized into three groups (never smoker, former smoker and current smoker). On the questionnaire, participants reported the type, duration and frequency of their participation in sports or recreational activities.^(25)^ When participants played any sport at least once a week, they were categorized as regular exercisers.^(26)^

### Definition of fatty liver

Fatty liver was diagnosed based on the results of abdominal ultrasonography, which was performed by trained technicians. All ultrasonographic images were stored in the image server as electric images, and gastroenterologists reviewed the images and made the diagnosis of fatty liver without reference to any of the participant’s other individual data. Of the four known criteria (hepatorenal echo contrast, liver brightness, deep attenuation and vascular blurring), the participants were required to have hepatorenal contrast and liver brightness to be given a diagnosis of fatty liver.^(23)^

### Statistical analysis

The study participants were divided into two groups based on the persistence or remission of NAFLD, and baseline characteristics were compared between the groups. Continuous variables were expressed as means (SD) and analyzed by Student’s *t* test. Categorical variables were expressed as numbers (percentages) and analyzed by Pearson’s chi-squared test. The hazard ratio of regular exercise for remission of NAFLD was calculated by Cox hazard model after adjusting for age, BMI, systolic blood pressure, fasting plasma glucose, high density lipoprotein (HDL) cholesterol, triglycerides, smoking states and alcohol consumption at baseline. *P* values less than 0.05 was considered statistically significant. JMP ver. 12.0 software (SAS Institute Inc., Cary, NC) was used for all statistical analyses.

## Results

**Fig. 1:**
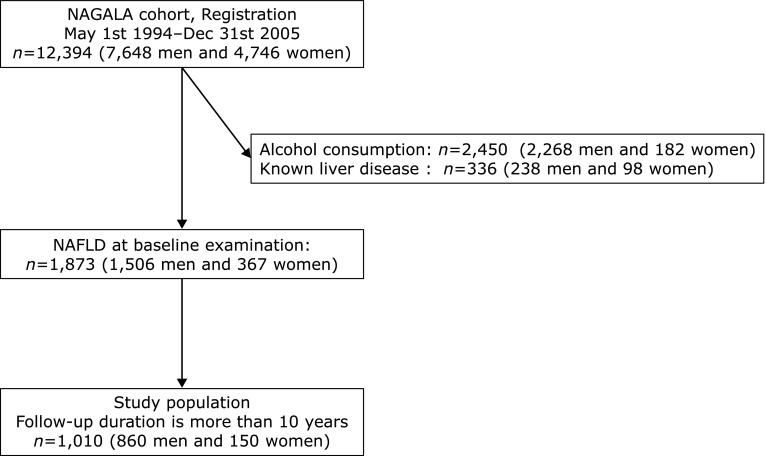
Inclusion and exclusion flow chart. NAGALA: NAfld in Gifu Area, Longitudinal Analysis.

From May 1, 1994 to Dec 31, 2005, the results of 12,394 participants (7,648 men and 4,746 women) were included in the NAGALA database 1994–2005 (Fig. 1). Among them, we excluded 2,450 participants (2,268 men and 182 women) because of alcohol consumption and 336 participants (238 men and 98 women) because of known liver disease. In addition, we also excluded 7,735 participants (3,636 men and 4,099 women) because they did not have NAFLD at baseline examination. Among them, 1,010 participants (860 men and 150 women) received health checkups for more than a decade.

The baseline characteristics of study participants are shown in Table 1. BMI of the participants with remission of NAFLD was lower than that of the participants with persistent NAFLD in both men and women. Among men, the percentage of regular exercisers in the participants with remission of NAFLD was higher than that in those with persistent NAFLD. In contrast, in women, the percentage of regular exercisers in the participants with remission of NAFLD was not different from those participants with persistent NAFLD.

**Fig. 2:**
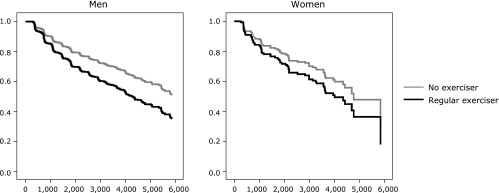
Hazard ratio of the effect of regular exercise on remission of NAFLD. The vertical axis is cumulative incidence of fatty liver and the horizontal axis is time as days. The black line represents regular exercisers. The gray line represents non-regular exercisers.

To clarify the effect of regular exercise on remission of NAFLD, we applied the Cox hazard model to calculate the hazard ratio of regular exercise for the remission of NAFLD after adjusting for age, BMI, fasting plasma glucose, systolic blood pressure, HDL cholesterol, triglycerides, smoking status, and alcohol consumption at baseline. Initially, the remission rate of NAFLD was higher in the regular exercisers (*p* = 0.015) for men but not for women (*p* = 0.430) (Fig. 2). Exercise habit as well as BMI were determinates for remission of NAFLD in men, with adjusted hazard ratios of 1.46 [95% confidence interval (95% CI) 1.10–1.95, *p* = 0.010] and 0.91 (95% CI 0.87–0.95, *p*<0.001), respectively. However, BMI was the only determinant for remission of NAFLD in women, with an adjusted hazard ratio of 0.86 (95% CI 0.77–0.95, *p* = 0.003) (Table 2).

## Discussion

In this cohort study, we showed clear evidence that regular exercise is associated with remission of NAFLD in men. Several cross-sectional studies revealed that regular exercise is associated with lower NAFLD prevalence.^(15–19)^ In addition, many randomized controlled trials revealed that exercise intervention is effective for reduction of liver fat.^(27–32)^ However, the sample sizes in these studies were small. Our study showed that regular exercise is associated with remission of NAFLD in men in a large-scale cohort study. The use of a validated, standardized ultrasonographic diagnosis of fatty liver, standardized questionnaires for lifestyle and the long duration of follow-up of more than a decade are the strengths of this cohort study.

The possible explanations for the effect of regular exercise on remission of NAFLD are as follows. The pathways of fatty acid oxidation and fatty acid synthesis have pivotal roles in hepatic steatosis. Adiponectin and adenosine monophosphate-activated protein kinase (AMPK) are associated with fatty acid oxidation in liver.^(33)^ AMPK is activated by adiponectin signaling.^(34)^ However, sterol regulatory element binding protein (SREBP)-1c enhances fatty acid synthesis.^(35)^ Exercise decreases triglyceride synthesis by increasing adiponectin^(36,37)^ and AMPK activation.^(38)^ In addition, exercise also reduces SREBP-1c levels in liver.^(39)^ Thus, exercise has a protective effect on fatty liver via activating the AMPK pathway and/or repressing the SREBP-1c pathway. Moreover it has been reported that exercise induced anti-oxidant enzymes via SIRT3.^(40)^

Our study revealed that the association between regular exercise and remission of NAFLD was observed in men but not in women. This might be because NAFLD was more common in men than in women, which is consistent with past reports.^(41)^ Therefore, the number of participants with NAFLD at baseline examination might not be enough to evaluate the effect of regular exercise on the natural history of NAFLD in women, because hazard ratios for regular exercise on remission of NAFLD were almost the same between men and women.

Some limitations of our study should be noted. First, ultrasonography may give an incorrect diagnosis compared to liver biopsy, although it has been validated for diagnosing fatty liver.^(20)^ However, it is impossible to perform liver biopsy in such a large number of healthy participants. Moreover, ultrasonography is a reasonable noninvasive surrogate measure for use in clinical settings and has a high sensitivity and specificity for diagnosing fatty liver.^(42)^ Second, self-reported information regarding exercise is frequently subject to misreporting, which could be a source of bias. Moreover, we did not measure the intensity of physical activity or the type of exercise, such as resistance training, aerobic training, or both. However, the purpose of this study was to determine the effect of regular exercise on remission of NAFLD. Third, this was a historical cohort study. Thus, we cannot exclude the possibility that our sample contained more health-concerned people than the general population of Japan. Forth, we don’t consider the influence of participant’s dietary.^(43,44)^ Lastly, the generalizability of our study to non-Japanese populations is uncertain.

To the best of our knowledge, this is the first study to investigate the association of remission of NAFLD and regular exercise with the natural history of NAFLD in a large-scale Japanese population. We suggest that regular exercise can remit NAFLD.

In conclusion, exercise at least once a week is implicated in the remission of NAFLD in men.

## Specific Author Contributions

Takafumi Osaka researched data, wrote manuscript and has approved the final draft submitted; Yoshitaka Hashimoto and Masahide Hamaguchi researched data, reviewed and edited the manuscript and has approved the final draft submitted; Takao Kojima and Akihiro Obora researched data and have approved the final draft submitted. Michiaki Fukui researched data, reviewed the manuscript and has approved the final draft submitted.

## Figures and Tables

**Table 1:** Baseline characteristics of the study participants

	Men				Women		
	Remission of NAFLD	Persistent of NAFLD	*p*^†^		Remission of NAFLD	Persistent of NAFLD	*p*^‡^
Total number	396 (46.0)	464 (54.0)	—		73 (48.6)	77 (51.4)	—
Age (years)	42.7 (7.1)	41.7 (7.0)	0.034		44.7 (7.6)	46.4 (6.8)	0.148
Body mass index (kg/m^2^)	24.8 (2.5)	26.0 (3.1)	<0.001		25.1 (2.7)	26.3 (3.1)	0.019
Systolic blood pressure (mmHg)	123.4 (15.1)	126.6 (14.6)	0.002		124.8 (17.2)	126.5 (18.8)	0.565
Diastolic blood pressure (mmHg)	77.9 (9.9)	79.6 (9.8)	0.012		77.2 (11.4)	78.0 (11.5)	0.687
Aspartate Aminotransferase (IU/L)	21.5 (8.5)	24.8 (10.6)	<0.001		18.2 (5.9)	20.0 (10.2)	0.187
Alanine aminotransferase (IU/L)	33.2 (16.8)	43.1 (24.4)	<0.001		21.7 (11.1)	27.3 (19.7)	0.035
γ-glutamyl-transferase (IU/L)	32.2 (26.4)	34.8 (24.2)	<0.001		18.6 (16.0)	24.5 (28.4)	0.123
Fasting plasma glucose (mmol/L)	5.7 (1.1)	5.7 (1.1)	0.522		5.7 (1.6)	5.5 (0.8)	0.327
HDL cholesterol (mmol/L)	1.1 (0.3)	1.0 (0.2)	0.356		1.3 (0.3)	1.3 (0.2)	0.384
Triglycerides (mmol/L)	1.9 (1.2)	1.9 (1.1)	0.624		1.5 (1.3)	1.4 (0.8)	0.549
Alcohol consumption (g/week)	42.7 (42.2)	37.8 (42.2)	0.114		15.2 (32.4)	11.8 (27.1)	0.512
Smoking states			0.795				0.97
Never smoker	133 (33.3)	162 (34.7)			66 (90.4)	69 (89.5)	
Former smoker	119 (30.2)	130 (28.1)			2 (2.7)	2 (2.6)	
Current smoker	144 (36.5)	172 (37.2)			5 (6.9)	6 (7.9)	
Physical activities							
Regular exerciser	68 (17.2)	52 (11.2)	0.012		9 (12.3)	6 (7.8)	0.355

Data are given as numbers (percentages), mean (SD). NAFLD, non-alcoholic fatty liver disease. HDL, high density lipoprotein. ^†^*p* value = remission of NAFLD vs persistent of NAFLD in men. ^‡^*p* value = remission of NAFLD vs persistent of NAFLD in women. ^†,‡^Student’s *t* test, Pearson’s qui-squared test were performed.

**Table 2:** Hazard ratio of baseline characteristics for the remission of NAFLD

	Men			Women	
	HR with 95% CI	*p*		HR with 95% CI	*p*
Age (years)	1.01 (0.99–1.03)	0.201		0.97 (0.94–1.01)	0.177
BMI (kg/m^2^)	0.91 (0.87–0.95)	<0.001		0.86 (0.77–0.95)	0.003
Alcohol consumption	1.00 (1.00–1.00)	0.215		1.01 (1.00–1.01)	0.134
Former smoker	0.98 (0.74–1.29)	0.984		0.77 (0.18–3.28)	0.464
Current smoker	0.98 (0.87–1.28)	0.984		0.57 (0.22–1.51	0.464
Regular exerciser	1.46 (1.10–1.95)	0.010		1.45 (0.66–3.16)	0.369
Fasting plasma glucose (mmol/L)	1.00 (0.98–1.02)	0.766		1.00 (0.98–1.02)	0.482
Systolic blood pressure (mmHg)	0.99 (0.98–1.00)	0.073		1.00 (0.99–1.02)	0.584
HDL cholesterol (mmol/L)	1.47 (1.00–2.15)	0.215		1.47 (0.46–3.14)	0.526
Triglycerides (mmol/L)	1.00 (0.84–1.09)	0.598		1.00 (0.86–1.11)	0.629

NAFLD, non-alcoholic fatty liver disease; HR, hazard ratio; CI, confidence interval; BMI, body mass index; HDL, high density lipoprotein. Hazard ratio of the regular exercise for the remission of NAFLD was calculated by Cox hazard model after adjusting by age, BMI, systolic blood pressure, fasting plasma glucose, HDL cholesterol, triglycerides, smoking status and alcohol consumption at baseline.

## References

[1] Neuschwander-Tetri BA, Caldwell SH (2003). Nonalcoholic steatohepatitis: summary of an AASLD Single Topic Conference. Hepatology.

[2] Angulo P (2002). Nonalcoholic fatty liver disease. N Engl J Med.

[3] Fukuda T, Hamaguchi M, Kojima T (2016). The impact of nonalcoholic fatty liver disease on incident type 2 diabetes mellitus in non-overweight individuals. Liver Int.

[4] Park SK, Seo MH, Shin HC, Ryoo JH (2013). Clinical availability of nonalcoholic fatty liver disease as an early predictor of type 2 diabetes mellitus in Korean men: 5-year prospective cohort study. Hepatology.

[5] Musso G, Gambino R, Tabibian JH (2014). Association of non-alcoholic fatty liver disease with chronic kidney disease: a systematic review and meta-analysis. PLoS Med.

[6] Hamaguchi M, Kojima T, Takeda N (2007). Nonalcoholic fatty liver disease is a novel predictor of cardiovascular disease. World J Gastroenterol.

[7] Akabame S, Hamaguchi M, Tomiyasu K (2008). Evaluation of vulnerable coronary plaques and non-alcoholic fatty liver disease (NAFLD) by 64-detector multislice computed tomography (MSCT). Circ J.

[8] Wong VW, Wong GL, Yip GW (2011). Coronary artery disease and cardiovascular outcomes in patients with non-alcoholic fatty liver disease. Gut.

[9] Hashimoto E, Taniai M, Tokushige K (2013). Characteristics and diagnosis of NAFLD/NASH. J Gastroenterol Hepatol.

[10] Hamaguchi M, Kojima T, Takeda N (2005). The metabolic syndrome as a predictor of nonalcoholic fatty liver disease. Ann Intern Med.

[11] Hamaguchi M, Takeda N, Kojima T (2012). Identification of individuals with non-alcoholic fatty liver disease by the diagnostic criteria for the metabolic syndrome. World J Gastroenterol.

[12] Liu H, Zhong H, Leng L, Jiang Z (2017). Effects of soy isoflavone on hepatic steatosis in high fat-induced rats. J Clin Biochem Nutr.

[13] Omagari K, Morikawa S, Nagaoka S (2009). Predictive factors for the development orregression of Fatty liver in Japanese adults. J Clin Biochem Nutr.

[14] Thoma C, Day CP, Trenell MI (2012). Lifestyle interventions for the treatment of non-alcoholic fatty liver disease in adults: a systematic review. J Hepatol.

[15] Kistler KD, Brunt EM, Clark JM, Diehl AM, Sallis JF, Schwimmer JB, NASH CRN Research Group. (2011). Physical activity recommendations, exercise intensity, and histological severity of nonalcoholic fatty liver disease. Am J Gastroenterol.

[16] Bae JC, Suh S, Park SE (2012). Regular exercise is associated with a reduction in the risk of NAFLD and decreased liver enzymes in individuals with NAFLD independent of obesity in Korean adults. PLoS One.

[17] Perseghin G, Lattuada G, De Cobelli F (2007). Habitual physical activity is associated with intrahepatic fat content in humans. Diabetes Care.

[18] Kantartzis K, Thamer C, Peter A (2009). High cardiorespiratory fitness is an independent predictor of the reduction in liver fat during a lifestyle intervention in non-alcoholic fatty liver disease. Gut.

[19] Noto H, Tokushige K, Hashimoto E, Taniai M, Shiratori K (2014). Questionnaire survey on lifestyle of patients with nonalcoholic steatohepatitis. J Clin Biochem Nutr.

[20] Hamaguchi M, Kojima T, Itoh Y (2007). The severity of ultrasonographic findings in nonalcoholic fatty liver disease reflects the metabolic syndrome and visceral fat accumulation. Am J Gastroenterol.

[21] Fukuda Y, Hashimoto Y, Hamaguchi M (2016). Triglycerides to high-density lipoprotein cholesterol ratio is an independent predictor of incident fatty liver; a population-based cohort study. Liver Int.

[22] Toshikuni N, Fukumura A, Hayashi N (2013). Comparison of the relationships of alcoholic and nonalcoholic fattyliver with hypertension, diabetes mellitus, and dyslipidemia. J Clin Biochem Nutr.

[23] McCullough AJ (2004). The clinical features, diagnosis and natural history of nonalcoholic fatty liver disease. Clin Liver Dis.

[24] Hashimoto Y, Hamaguchi M, Kojima T (2015). The modest alcohol consumption reduces the incidence of fatty liver in men: a population based large scale cohort study. J Gastroenterol Hepatol.

[25] Aaron DJ, Kriska AM, Dearwater SR, Cauley JA, Metz KF, LaPorte RE (1995). Reproducibility and validity of an epidemiologic questionnaire to assess past year physical activity in adolescents. Am J Epidemiol.

[26] Ryu S, Chang Y, Kim DI, Kim WS, Suh BS (2007). γ-Glutamyltransferase as a predictor of chronic kidney disease in nonhypertensive and nondiabetic Korean men. Clin Chem.

[27] Bacchi E, Negri C, Targher G (2013). Both resistance training and aerobic training reduce hepatic fat content in type 2 diabetic subjects with nonalcoholic fatty liver disease (the RAED2 Randomized Trial).. Hepatology.

[28] Keating SE, Hackett DA, Parker HM (2015). Effect of aerobic exercise training dose on liver fat and visceral adiposity. J Hepatol.

[29] Zelber-Sagi S, Buch A, Yeshua H (2014). Effect of resistance training on non-alcoholic fatty-liver disease a randomized-clinical trial. World J Gastroenterol.

[30] Sun W-H, Song M-Q, Jiang C-Q (2012). Lifestyle intervention in non-alcoholic fatty liver disease in Chengyang District, Qingdao, China. World J Hepatol.

[31] Haus JM, Solomon TPJ, Kelly KR (2013). Improved hepatic lipid composition following short-term exercise in nonalcoholic fatty liver disease. J Clin Endocrinol Metab.

[32] Oh S, Shida T, Yamagishi K (2015). Moderate to vigorous physical activity volume is an important factor for managing nonalcoholic fatty liver disease: a retrospective study. Hepatology.

[33] Senmaru T, Fukui M, Okada H (2013). Testosterone deficiency induces markedly decreased serum triglycerides, increased small dense LDL, and hepatic steatosis mediated by dysregulation of lipid assembly and secretion in mice fed a high-fat diet. Metabolism.

[34] Yamauchi T, Kamon J, Minokoshi Y (2002). Adiponectin stimulates glucose utilization and fatty-acid oxidation by activating AMP-activated protein kinase. Nat Med.

[35] Méndez-Sánchez N, Arrese M, Zamora-Valdés D, Uribe M (2007). Current concepts in the pathogenesis of nonalcoholic fatty liver disease. Liver Int.

[36] Kriketos AD, Gan SK, Poynten AM, Furler SM, Chisholm DJ, Campbell LV (2004). Exercise increases adiponectin levels and insulin sensitivity in humans. Diabetes Care.

[37] Huang H, Iida KT, Sone H, Yokoo T, Yamada N, Ajisaka R (2006). The effect of exercise training on adiponectin receptor expression in KKAy obese/diabetic mice. J Endocrinol.

[38] Park H, Kaushik VK, Constant S (2002). Coordinate regulation of malonyl-CoA decarboxylase, sn-glycerol-3-phosphate acyltransferase, and acetyl-CoA carboxylase by AMP-activated protein kinase in rat tissues in response to exercise. J Biol Chem.

[39] Cintra DE, Ropelle ER, Vitto MF (2012). Reversion of hepatic steatosis by exercise training in obese mice: the role of sterol regulatory element-binding protein-1c. Life Sci.

[40] Tsukiyama Y, Ito T, Nagaoka K, Eguchi E, Ogino K (2017). Effects of exercise training on nitric oxide, blood pressure and antioxidant enzymes. J Clin Biochem Nutr.

[41] Omagari K, Kadokawa Y, Masuda J (2002). Fatty liver in non-alcoholic non-overweight Japanese adults: incidence and clinical characteristics. J Gastroenterol Hepatol.

[42] Hernaez R, Lazo M, Bonekamp S (2011). Diagnostic accuracy and reliability of ultrasonography for the detection of fatty liver: a meta-analysis. Hepatology.

[43] Mitsuhashi K, Hashimoto Y, Tanaka M (2017). Combined effect of body mass index and waist-height ratio on incident diabetes; a population based cohort study. J Clin Biochem Nutr.

[44] Miki A, Hashimoto Y, Tanaka M (2017). Urinary pH reflects dietary acid load in patients with type 2 diabetes. J Clin Biochem Nutr.

